# Systemic Lupus Erythematosus Complicated by Spontaneous Splenic Rupture

**DOI:** 10.7759/cureus.95910

**Published:** 2025-11-01

**Authors:** Li Wang, Qianyu Zhou, Feng Liu, Peng Chen, Mingdao Hu, Haixia Tan

**Affiliations:** 1 Hepatopancreatobiliary Surgery, The Second Affiliated Hospital of Kunming Medical University, Kunming, CHN

**Keywords:** asr, non-traumatic splenic rupture, sle, ssr, systemic lupus erythematosus

## Abstract

Spontaneous splenic rupture (SSR) is an uncommon but life-threatening surgical emergency. When it occurs as a complication of systemic lupus erythematosus (SLE), the diagnostic and therapeutic strategy becomes particularly complex. Published literature on SSR in the setting of SLE is sparse, and there is no clear consensus on the optimal management of such a surgical catastrophe. What is the optimal treatment strategy for patients with SSR complicated by SLE? This paper addresses these questions by providing an in-depth analysis of a representative case of SLE complicated by SSR that we successfully diagnosed and treated.

Our case study details the patient’s entire clinical course, from the initial presentation with non-specific abdominal pain, through confirmation via imaging diagnostics, to the final recovery achieved through a personalized treatment plan developed by a multidisciplinary team (MDT).

A key finding of our research is the emphasis that, for patients with known autoimmune diseases such as SLE, the acute onset of abdominal pain, hemodynamic instability, or any signs of intra-abdominal bleeding should prompt clinicians to maintain a high index of suspicion and include SSR in the differential diagnosis. Rapid identification, multidisciplinary collaboration (involving close coordination among rheumatology, critical care, surgery, and radiology), and individualized treatment decisions based on the patient’s specific condition are the cornerstones of successfully managing this severe complication and improving patient outcomes.

We believe the significance of our findings lies in their potential to greatly enhance clinicians’ understanding of this rare yet fatal complication of SLE. The detailed diagnostic and therapeutic pathway and the decision-making rationale provided in this report can serve as a valuable reference guide for colleagues worldwide when faced with similar challenging cases, potentially saving more patients’ lives.

## Introduction

Systemic lupus erythematosus (SLE) is a multisystem autoimmune disease characterized by immune-mediated inflammatory responses, the presence of various autoantibodies, and multi-organ involvement. Acute abdominal complications of SLE include abdominal crises such as acute gastroenteritis and spontaneous rupture of the liver or spleen, among others. These complications may be related to immune complex deposition in abdominal vasculitis and abdominal vascular embolism.

Splenic rupture can be traumatic or spontaneous (spontaneous splenic rupture, SSR, or acute splenic rupture, ASR), and is often caused by malignant tumors, hematologic diseases, splenic infarction, or drastic changes in intra-abdominal pressure [1]. SSR is an extremely rare complication of SLE, with only a limited number of cases reported globally. Diagnostic delays can lead to historically high mortality rates, with some literature series reporting mortality exceeding 50% in previous studies. This case report aims to improve clinicians’ awareness of SSR in patients with SLE.

## Case presentation

A 48-year-old female with a past medical history of SLE and cerebellar infarction, who presented with dizziness, was admitted to the hospital’s Department of Rheumatology and Immunology due to five days of abdominal pain. The pain was localized to the upper abdomen, moderate in severity, and exacerbated by eating. The patient vomited a small amount of gastric contents without coffee-ground material. She had been diagnosed with the disease for over three years and was currently taking cyclosporine A (50 mg twice daily) and prednisone acetate tablets (15 mg three times daily) for treatment. Physical examination revealed pale palpebral conjunctiva and tenderness in the left upper abdomen, with no rebound tenderness or muscle tension. The patient’s vital signs were as follows: temperature (T), 36.5°C; pulse (P), 84 beats per minute; respiration (R), 21 breaths per minute; blood pressure (BP), 109/75 mmHg; and oxygen saturation (SpO₂), 99%.

**Figure 1:**
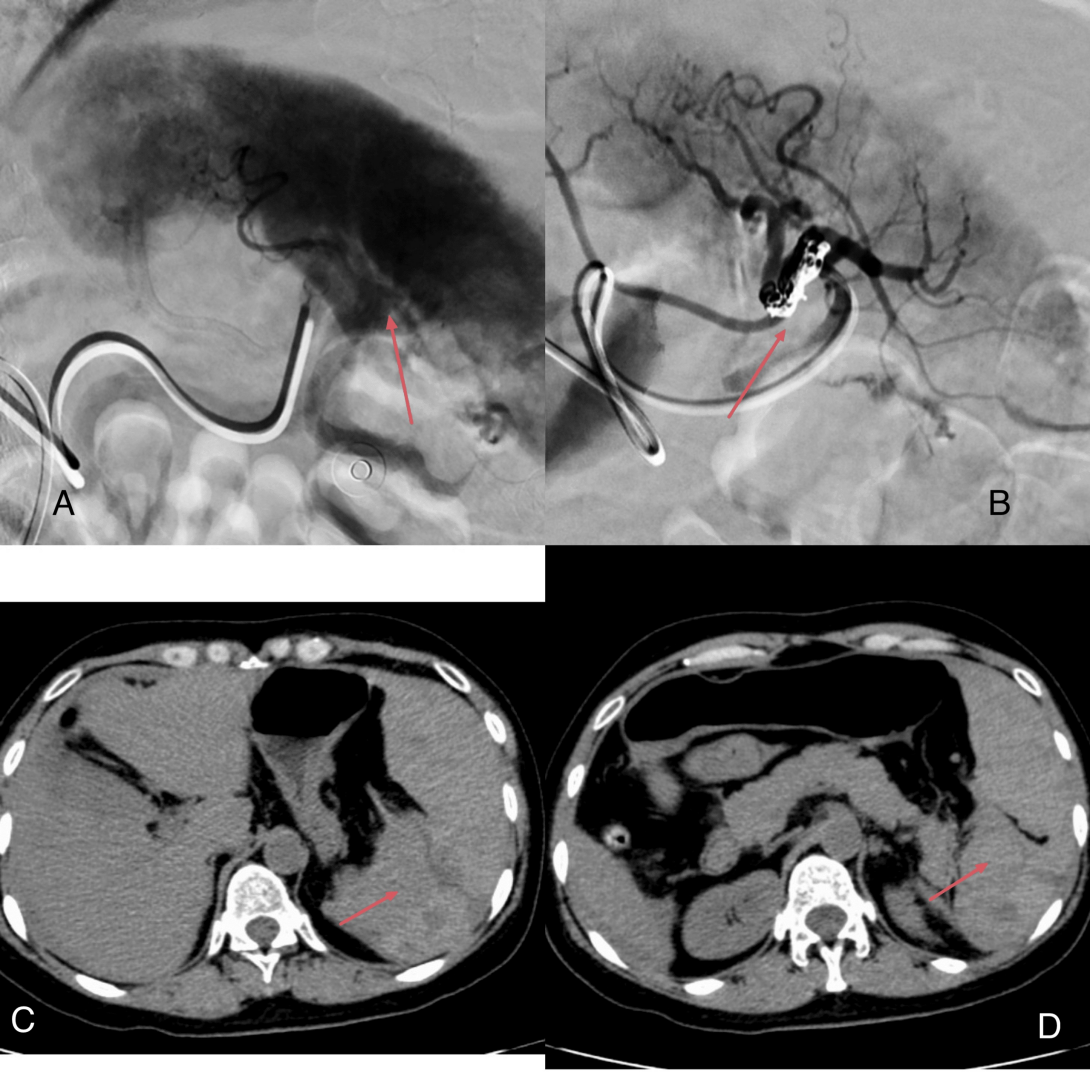
Pre- and post-embolization imaging of splenic rupture. (A) Pre-procedural angiogram shows active contrast extravasation (arrow) from the spleen. (B) Post-embolization angiogram confirms successful occlusion of the bleeding vessel. (C, D) Pre-operative emergency computed tomography (CT) scans reveal an irregular splenic contour with a perisplenic hematoma. Combined with the patient’s decreased hemoglobin levels, these findings suggest the presence of a hematoma.

Abdominal ultrasound showed a small amount of fluid around the spleen. CT imaging revealed an irregular mixed-density mass shadow in the pancreaticogastric-splenic space and fluid accumulation in the abdominal and pelvic cavities, suggesting possible intra-abdominal hemorrhage or ascites. Corresponding angiographic images demonstrate active splenic bleeding and post-embolization occlusion (Figure 1). The CBC and routine biochemical test results are shown in Table 1. The patient was referred to the Department of Hepatobiliary and Pancreatic Surgery due to concerns about splenic rupture. After bedside consultation, the preliminary diagnoses were splenic rupture and hemorrhagic shock. At that time, her vital signs were: T, 36.6°C; P, 95 beats per minute; R, 22 breaths per minute; BP, 100/60 mmHg; and SpO₂, 99%.

**Table 1:** Changes in hematological and biochemical parameters during hospitalization. Table 1 presents the laboratory parameters (complete blood count and biochemical profile) of the patient at admission, pre-intervention, post-intervention, and pre-discharge. Not all parameters were rechecked at every stage; therefore, unmeasured or unrepeated items are not listed.

Parameter Category	Test	On Admission	Pre-Intervention	Post-Intervention	Before Discharge	Reference Range	Unit
Blood Cell Analysis	Leukocyte count	9.62	16.36	13.75	9.88	4-10	×10⁹/L
	Platelet count (PLT)	62	77	108	89	150-400	×10⁹/L
	Hemoglobin (Hb)	84	47	53	88	120-160	g/L
Biochemical Parameters	Liver Function Tests						
	Albumin (ALB)	35.4	-	35.6	33.4	35-50	g/L
	Alanine aminotransferase (ALT)	31	-	21	23	10-40	U/L
	Aspartate aminotransferase (AST)	20	-	74	15	10-40	U/L
	Total bilirubin (TBIL)	13.7	-	19.9	18.8	5.1-20.5	μmol/L
Renal Function Tests	Creatinine (Cr)	208	-	81	78	53-97	μmol/L
	Urea (Carbamide)	13.56	-	10.58	4.21	2.5-7.1	mmol/L
	Glomerular filtration rate (GFR)	24	-	75	78	>90	mL/min
	Amylase (AMY)	157	-	-	-	30-100	U/L
	Lipase (LIP)	122	-	-	-	<60	U/L

Exploratory laparotomy was recommended; however, the patient refused surgical intervention and opted for conservative management. She received symptomatic treatment, including rapid intravenous infusions of 500 ml of 0.9% sodium chloride injection, 500 ml of 5% glucose injection, and 500 ml of sodium acetate Ringer’s injection within a short period for anti-shock therapy. Despite these interventions, her condition did not significantly improve. The rechecked test results are shown in Table 1, and biochemical tests were not repeated. Her updated vital signs were: T, 36.6°C; P, 116 beats per minute; R, 25 breaths per minute; BP, 85/61 mmHg; and SpO₂, 99%. 

## Discussion

According to the diagnostic criteria established by Orloff MJ and Peskin GW in 1958, SSR is defined as the rupture of the spleen in the absence of significant trauma or with only minor traumatic injury [2-3]. It is a rare abdominal emergency, often associated with infectious, hematologic, neoplastic, and autoimmune diseases. This case report describes an instance of SSR triggered by SLE, an autoimmune disorder, representing a rare clinical scenario with complex and noteworthy pathophysiology.

The pathophysiological mechanisms underlying SSR in SLE have not been fully elucidated, but several contributing factors are currently recognized. First, autoantibodies and immune complexes produced in SLE patients can deposit in the walls of splenic blood vessels, inducing vasculitis, increasing vascular fragility, and leading to necrosis and potential rupture [3]. Second, immune-mediated vasculitis may cause splenic microinfarctions. The softened tissue in infarcted areas forms vulnerable foci that may rupture even under normal portal venous pressure or minor stressors such as vomiting or coughing [4]. Furthermore, active SLE is frequently complicated by thrombocytopenia, as seen in this patient (platelet count: 62 × 10⁹/L), which exacerbates bleeding tendency and impairs spontaneous hemostasis following rupture.

The diagnosis and treatment process of this case offer valuable insights. The patient primarily presented with abdominal pain and severe anemia (hemoglobin: 49 g/L). Imaging studies (CT) revealed a perisplenic mass and ascites, highly suggestive of splenic rupture and hemorrhage. Although laparotomy or splenectomy remains the traditional gold standard for managing splenic rupture, especially in hemodynamically unstable patients, due to the patient’s refusal of surgery and relatively stable condition, a more individualized approach was adopted: selective embolization of the bleeding branches of the splenic artery. This is a case report of a patient who experienced hemorrhagic shock, was stabilized after resuscitation, and subsequently underwent successful interventional embolization. The case highlights the procedure’s value even in patients who are hemodynamically stable following initial resuscitation. It not only precisely occluded the bleeding site and effectively controlled hemorrhage but also maximally preserved healthy splenic tissue and its immune function, which is particularly crucial for SLE patients at long-term risk of infection [5]. Subsequently, the continuous rise in the patient’s hemoglobin strongly confirmed the efficacy and safety of this treatment modality.

However, this case has certain limitations. As a single-case report, it precludes large-scale statistical analysis. Moreover, the systemic lupus erythematosus disease activity index (SLEDAI) was not thoroughly assessed during this acute event, making it difficult to precisely quantify the correlation between SLE activity and the occurrence of SSR.

## Conclusions

In summary, SLE complicated by SSR is a rare but life-threatening abdominal emergency and should always be included in the differential diagnosis. Management should involve a MDT approach to ensure individualized treatment planning. Notably, selective splenic artery embolization represents an effective treatment option that preserves splenic function, offering the advantages of minimal invasiveness and faster recovery compared to splenectomy. Further research is needed to elucidate the underlying mechanisms and to compare the efficacy of different treatment modalities in order to establish standardized management pathways.
